# Synovium is a sensitive tissue for mapping the negative effects of systemic iron overload in osteoarthritis: identification and validation of two potential targets

**DOI:** 10.1186/s12967-023-04541-5

**Published:** 2023-09-23

**Authors:** Zhuangzhuang Jin, He Zhang, Lunhao Bai, Lingyu Yue, Weiming Zhang, Jiajian Liang, Bohan Chang, Yue Yang, Zhehan Hu, Liang Chen, Chuanji Guo

**Affiliations:** 1 Department of Emergence Medicine, Shengjing Hospital of China Medical University, Shenyang, Liaoning China; 2 Department of Bone and Soft Tissue Oncology, Shengjing Hospital of China Medical University, Shenyang, Liaoning China; 3 Department of Orthopedic Surgery, Shengjing Hospital of China Medical University, Shenyang, Liaoning China; 4 Beijing AnDing Hospital of Capital Medical University, Beijing, China; 5 Department of Rheumatology, The First Affiliated Hospital of China Medical University, Shenyang, Liaoning China; 6 Hospital Administration Office, Shengjing Hospital of China Medical University, No. 36, Sanhao Street, Heping District, Shenyang City, Liaoning Province China

**Keywords:** Bioinformatics analysis, Iron overload, Osteoarthritis, Single-cell RNA-seq analysis, Synovium, Validation experiments

## Abstract

**Background:**

The prevention and treatment of osteoarthritis (OA) pose a major challenge in its research. The synovium is a critical tissue in the systematic treatment of OA. The present study aimed to investigate potential target genes and their correlation with iron overload in OA patients.

**Methods:**

The internal datasets for analysis included the microarray datasets GSE46750, GSE55457, and GSE56409, while the external datasets for validation included GSE12021 and GSE55235. The GSE176308 dataset was used to generate single-cell RNA sequencing profiles. To investigate the expression of the target genes in synovial samples, quantitative reverse transcription-PCR, western blotting, and immunohistochemical assay were conducted. ELISA was used to detect the levels of ferritin and Fe^2+^ in both serum and synovium.

**Results:**

*JUN* and *ZFP36* were screened from the differentially expressed genes, and their mRNA were significantly reduced in the OA synovium compared to that in normal synovium. Subsequently, complex and dynamically evolving cellular components were observed in the OA synovium. The mRNA level of *JUN* and *ZFP36* differed across various cell clusters of OA synovium and correlated with immune cell infiltration. Moreover, ferritin and Fe^2+^ were significantly increased in the serum and synovium of OA patients. Further, we found that *JUN* elevated and *ZFP36* decreased at protein level.

**Conclusions:**

The synovium is a sensitive tissue for mapping the adverse effects of systemic iron overload in OA. *JUN* and *ZFP36* represent potential target genes for attenuating iron overload during OA treatment. Some discrepancies between the transcription and protein levels of *JUN* suggest that post-transcriptional modifications may be implicated. Future studies should also focus on the roles of *JUN* and *ZFP36* in inducing changes in cellular components in the synovium during OA pathogenesis.

**Supplementary Information:**

The online version contains supplementary material available at 10.1186/s12967-023-04541-5.

## Introduction

Osteoarthritis (OA) is a degenerative and heterogeneous disease with a current worldwide incidence of more than 240 million people, and it may deteriorate with age [1]. The conventional belief that cartilage serves as the fundamental basis to diagnose and treat OA has been extended following advances in clinical research [2]. Considering the interaction between the synovium and joint cartilage, the synovium deserves major attention in research on the pathogenesis and treatment of OA. The synovium is the main source of joint fluid with a unique and crucial biological function of cultivating, maintaining, and protecting chondrocytes [3]. The synovium also serves as a relay station for communication between the blood and joint cavity and facilitates the conversion of systemic inflammation into OA-specific inflammation [4]. In patients with OA, the synovium shows synovial lining hyperplasia, fibrosis, and stromal vascularization, which may be responsible for the pain and swelling associated with OA symptoms [5]. Consequently, the synovium is highly relevant in therapeutic strategies for OA. However, the etiology of synovitis development and its interaction with joint cartilage throughout OA progression remains a debatable topic.

Iron, an essential trace element, is crucial for multiple metabolic processes in cell function and fate [6]. Iron is increasingly recognized as a key contributor to aging-related pathologies because of its proclivity to catalyze the generation of free radicals [7]. Iron homeostasis in serum and cells is precisely modulated to maintain optimal biological functioning through iron acquisition, storage, and efflux [8]. Recent studies have increasingly supported the prominent role of iron overload in OA progression. These studies indicate the presence of an increased amount of iron ions in the synovial fluid and deposited iron in the synovium of OA patients [9, 10]. Additionally, a positive correlation was observed between the increased ferritin level and OA deterioration [11]. Thus, the synovium plays a pivotal role in linking systemic iron overload with joint iron overload. Nevertheless, the precise pathophysiology of iron overload in OA-associated synovitis remains incompletely understood.

Because of its diverse cellular components and responses, the synovium induces M1 polarization of macrophages, neutrophil enrichment, and fibroblast proliferation, thus rendering it susceptible to iron overload [12–14]. Iron overload triggers several adverse events, including the abundant production of reactive oxygen species, failure of the antioxidant defense system, metabolic disorders, and cellular ferroptosis [15]. OA-associated synovitis and iron overload share similar characteristics such as oxidative stress and lipid peroxidation [16, 17]. Moreover, previous studies have demonstrated that targeting iron overload or ferroptosis can be a feasible approach to treat OA [18, 19]. It is therefore imperative to conduct a more comprehensive examination of genes that exhibit targeting potential or differential expression, which could be used in mitigating iron overload during OA treatment.

In the present study, we screened potential target genes from the differentially expressed genes (DEGs) and investigated their correlations with iron overload by using data from a public database. We also assessed the expression and relevance of these potential targets in various cell clusters of the synovium by CIBERSORT immune infiltration analysis and single-cell RNA sequencing (RNA-seq) profiling. Subsequently, we detected iron load, adverse events, and the expression of the potential target genes in the clinical samples of 12 patients with OA of varying severity. The present study provided insights into the potential targets for the diagnosis and treatment of OA by alleviating iron overload.

## Materials and methods

### Microarray gene data collection and data processing

**Fig. 1:**
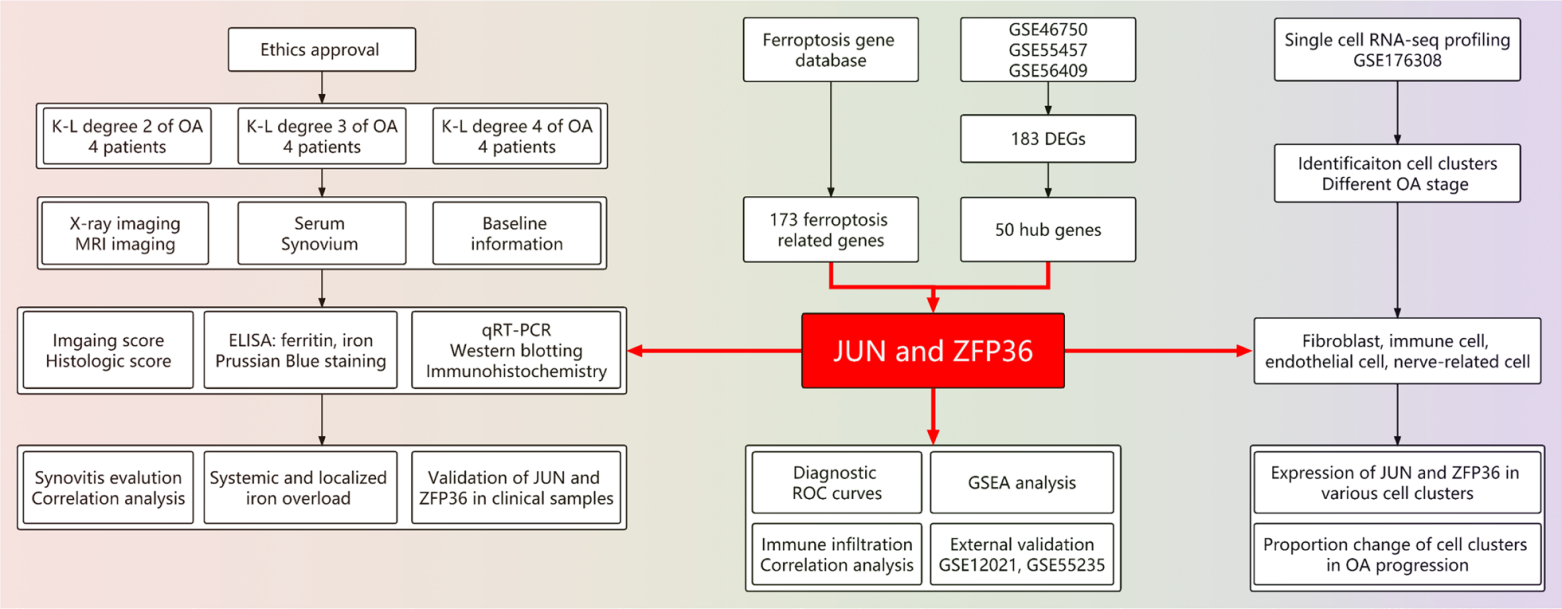
Flowchart of the validation experiment, bioinformatics analysis, and single-cell RNA-seq profiling

Microarray data of synovium samples were collected from the GEO database (https://www.ncbi.nlm.nih.gov/geo/), including GSE56409, GSE46750, and GSE55457 as the internal datasets for analysis and GSE12021 and GSE55235 as the external datasets for validation. The MINiML formatted family files contained information on raw data from all platforms, samples, and GSE records. The internal dataset contained 27 normal and 27 OA samples. The external dataset comprised 23 control group (CG) and 30 OA samples. Prior to differential expression analysis, raw gene expression profiles were normalized by log2 transformation using the preprocessCore package in R software (version 4.1.3). Batch effects were corrected using the removeBatchEffect function from the limma package. A principal component analysis (PCA) plot was generated using the original expression values with the ggord package to confirm and evaluate the variability present in the dataset. A total of 173 ferroptosis-related genes were obtained from the FerrDb database (http://www.zhounan.org/ferrdb/current/). Figure 1 shows the flowchart depicting the experimental design used in this study.

### Differential gene expression and pathway enrichment analysis

The limma package in R software was used to detect DEGs. Adjusted *P* < 0.05 and |log(fold change)|> 1.5 were used as the threshold criteria for the DEGs. We generated a heat map to visualize the expression of the top 50 DEGs by using the pheatmap package in R software. The Gene Ontology (GO) functional enrichment analysis and the Kyoto Encyclopedia of Genes and Genomes (KEGG) pathway enrichment analysis were conducted using the Clusterprofiler package (version 3.18.0) according to the classification of both upregulated and downregulated DEGs.

### Protein–protein interaction network and identification of hub genes

The protein–protein interaction (PPI) network was constructed with synovitis-related DEGs obtained from the above-mentioned internal dataset to identify the most critical genes (hub genes). We used the STRING database (http://string-db.org/) to predict the PPI network of the DEGs based on experimental evidence and information regarding *Homo sapiens* from other databases [20]. A combined score of more than 0.4 was considered the cutoff criterion, and the Cytoscape software (version 3.9.1) was used to visualize the PPI network [21].

### Receiver operating characteristic analysis

Receiver operating characteristic (ROC) analysis was performed using the pROC package (version 1.17.0.1) to determine the area under the curve (AUC) and confidence intervals.

### Gene set enrichment analysis

Gene set enrichment analysis (GSEA) was performed using GSEA v3.0 (http://www.broadinstitute.org/gsea). Gene sets were obtained from MSigDB (http://www.gsea-msigdb.org/gsea/downloads.jsp) to evaluate the related pathways and molecular mechanisms. Significance was considered at a *P*-value of < 0.05 and a false discovery rate (FDR) of < 0.25.

### Single-cell RNA-seq profiles

The single-cell RNA-seq profile used in the present study is a subset of the single-cell RNA-seq published by Nanus et al. and is publicly available at the GEO series GSE176308 [22]. Briefly, the Seurat package was used to filter out low quality cells based on the following two quality measures: number of genes detected < 200 or > 6000 and mitochondrial content > 10%. Corrected-normalized data metrics were applied to the standard analysis. The Uniform Manifold Approximation and Projection (UMAP) method was used to visualize and cluster the principal components. Subpopulations were named according to the top 10 marker genes. The expression of the target genes in each cluster was calculated and visualized.

### Patient information and inclusion–exclusion criteria

The present study was approved by the ethics committee of Shengjing Hospital (Approval No. 2022PS1014K). Written informed consent was obtained from all patients. The clinical information and imaging data were obtained from the hospital information system of Shengjing Hospital affiliated to China Medical University.

The inclusion criteria were as follows: age ≥ 50 years, preoperative radiography and MRI performed on the knee, radiographic signs of knee degeneration, a natural history of knee degeneration, and hospitalization for knee arthroplasty or knee arthroscopy. The exclusion criteria were as follows: history of severe trauma or surgery of the knee, tumor or infection of the knee, risk factors that might exacerbate the degeneration process (nicotine/tobacco addiction, corticosteroid therapy in the preceding 6 months, chemotherapy, or other factors), situations in which sample acquisition could affect patient outcomes, and refusal of informed consent.

To fully respect the patient’s right to informed consent, we explained to patients or their authorized persons the timing and size of the synovium samples to be obtained during the surgery. Every patient had the right to withdraw from the experiment at any time and could request the destruction of samples and related experimental data.

### Sample collection and preservation conditions

The synovial samples were collected from the suprapatellar capsule to avoid iatrogenic injury. The samples were then stored in a refrigerator at − 80 °C or fixed with 4% paraformaldehyde for the subsequent experiments.

### Hematoxylin–eosin staining

Staining with hematoxylin–eosin (HE) (G1120, Solarbio, China) was performed as follows. Briefly, paraffin-embedded synovial sections were deparaffinized and rehydrated with a graded series of xylene and ethanol. The sections were stained with hematoxylin for 10 min and differentiated with 1% hydrochloric alcohol for 10 s. The sections were then immersed in eosin for 10 min. Finally, the sections were observed under an optical microscope for histopathological evaluation.

### Prussian Blue staining

Briefly, the sections were stained with a Prussian Blue solution (G1422, Solarbio, China) for 1.5 h. The sections were then counterstained with Nuclear Fast Red. After dehydration and mounting, the sections were observed and evaluated under an optical microscope.

### Immunohistochemistry

The sections were dewaxed, and antigen repair was performed with an enzymatic antigen retrieval solution (AR0026, Boster Biological Technology, USA) at 37 °C for 30 min. Endogenous peroxidase activity was quenched using 0.3% H_2_O_2_ for 1 h. Next, 5% goat serum was added to block the nonspecific binding sites. The sections were then incubated with the following primary antibodies overnight at 4 °C: rabbit polyclonal anti-*JUN* (1:100; 24,909-1-AP, Proteintech, China), rabbit polyclonal anti-*ZFP36* (1:100; 12,737-1-AP, Proteintech), rabbit polyclonal anti-*GPX4* (1:100, abs136221, Absin). Subsequently, the sections were rinsed thrice with phosphate-buffered saline for 5 min. The sections were then sequentially incubated with biotin-conjugated secondary antibodies and horseradish peroxidase (HRP)-conjugated secondary antibodies at 22 °C ± 2 °C for 30 min. The immunoreactivity was visualized by diaminobenzidine, while nuclear staining was performed with hematoxylin. The relative expression level of the target protein was calculated using Image-Pro Plus version 6.0 software (Media Cybernetics, Rockville, MD, USA).

### Quantitative reverse transcription-PCR

Briefly, total RNA was extracted from the synovial samples by using an AG RNAex Pro Reagent (AG21102, Accurate Biology, China). cDNA was synthesized from the extracted RNA by using a 5 × Evo M-MLV RT Reaction Mix (AG11728, Accurate Biology). A 2 × SYBR^®^ Green Pro Taq HS Premix (AG11701, Accurate Biology) and a real-time fluorescence quantitative PCR instrument (FQD-96A, Bioer Technology Co., Ltd., China) were used for the qRT-PCR assay. The relative expression of the target genes was calculated using the 2^−ΔΔCT^ method. *GAPDH* was used as an internal control. The primers used for qRT-PCR were designed and synthesized by Huizhi Tongda Biological Science Co., Ltd (Shenyang, China). Table 1 shows detailed information of the primers.

**Table 1:** Sequences of primers used in the study

Primer name	Primer sequence (5′–3′)
Human-*GAPDH* forward primer	AGAAGGCTGGGGCTCATTTG
Human-*GAPDH* reverse primer	AGGGGCCATCCACAGTCTTC
*JUN* forward primer	AGCGCCTGATAATCCAGT
*JUN* reverse primer	TCCTGCTCATCTGTCACGTT
*ZFP36* forward primer	AGCCTGACTTCAGCGCTCC
*ZFP36* reverse primer	GCGACAGGAGGCTCTCGTA

### Western blotting assay

Briefly, the whole tissue protein of synovial samples was extracted using RIPA lysis buffer (P0013C, Beyotime, China) containing 1% PMSF (ST506, Beyotime, China). The supernatant was collected and quantified using the BCA protein assay kit (abs9232, Absin, China). Equivalent quantities of proteins (30 μg) were electrophoresed on a 10% SDS/polyacrylamide gel and electroblotted onto polyvinylidene difluoride (PVDF) membranes. Nonspecific binding sites were blocked with a rapid blocking solution (PS108P, Epizyme Biotechnology, China). The membranes were incubated with the following primary antibodies overnight at 4 °C: rabbit polyclonal anti-*JUN* (1:1000; 24909-1-AP, Proteintech), rabbit polyclonal anti-*ZFP36* (1:1000; 12737-1-AP, Proteintech), rabbit polyclonal anti-*GPX4* (1:1000, abs136221, Absin), and rabbit polyclonal anti-*GAPDH* (1:20000; 10494-1-AP, Proteintech). The membranes were washed three times with TBST and then incubated with IgG-HRP-conjugated secondary antibodies at room temperature for 2 h. Chemiluminescence was detected with an enhanced chemiluminescence reagent. The protein bands were scanned and analyzed using the Image-Pro Plus version 6.0 software according to the grayscale value. *GAPDH* was used as the internal control.

### ELISA

The levels of ferritin and Fe^2+^ in serum and synovial samples were measured using ELISA kits (Mlbio, China) in accordance with the manufacturer’s instructions.

### Statistical analysis

The normality and homogeneity of variance were assessed using the Shapiro–Wilk test and Levene test, respectively. Statistical significance was determined using one-way analysis of variance. The nonparametric Kruskal–Wallis test was used for non-normally distributed data. Statistical analyses were conducted using SPSS version 21.0 (IBM Corp., Armonk, NY, USA). The significance level was set at *P* < 0.05.

## Results

### *JUN* and *ZFP36* were identified as hub genes in the development of OA-associated synovitis

**Fig. 2:**
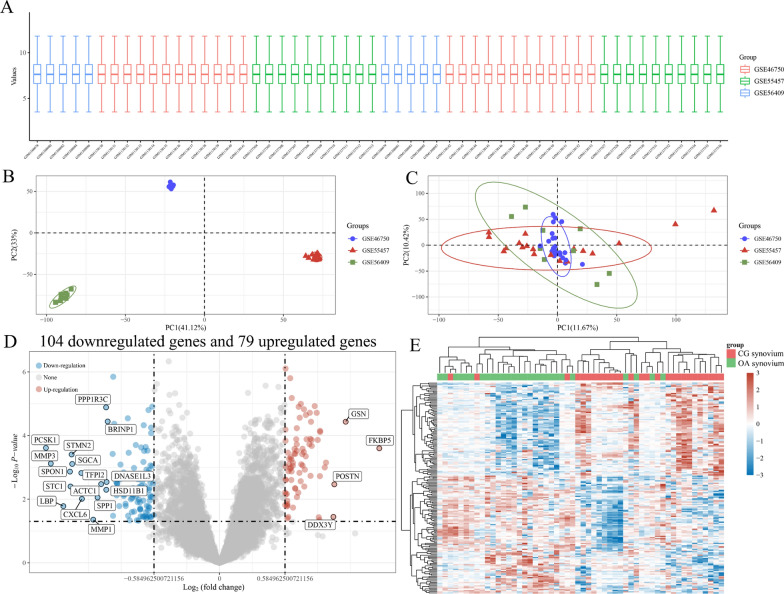
Differentially expressed genes and results of related bioinformatics analyses. **A** Boxplot of the normalized data. **B** PCA results before batch effect removal for multiple datasets. **C** PCA results after batch effect removal for multiple datasets. **D** Volcano plot of differentially expressed genes. **E** Heatmap of differentially expressed genes. PCA, principal component analysis

The linear distribution of box plots indicated the data homogeneity of the internal datasets (GSE46750, GSE55457, and GSE56409) (Fig. 2A). After excluding batch effects, the datasets showed an overlap, thus indicating their suitability for further analysis as a merged batch of data, following independent distribution prior to de-batching effects (Fig. 2B and C). A total of 183 DEGs (104 upregulated and 79 downregulated) were identified, as shown in the volcano plots (Fig. 2D). The generated heatmap shows an overall view of the largest magnitude expression change of the top 50 DEGs (Fig. 2E). The KEGG pathway enrichment analysis indicated that the DEGs were mainly enriched in “TNF pathway,” “MAPK pathway,” etc. The GO functional enrichment analysis showed that the DEGs were most uniquely enriched in terms such as “immune effector process,” “ossification,” and “RNA stability” (Additional file 1: Enrichment analysis of KEGG and GO).

**Fig. 3:**
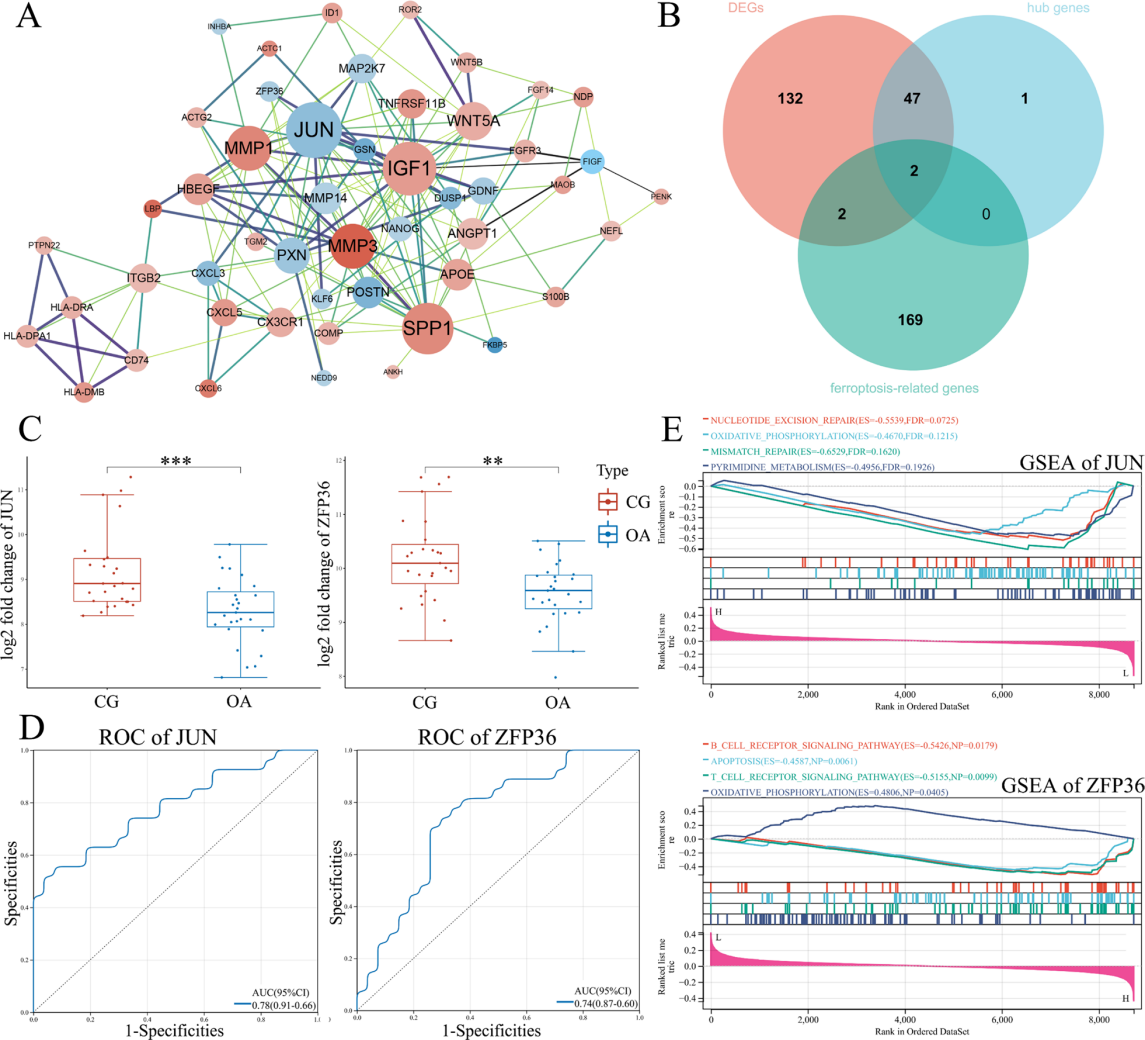
Identification of *JUN* and *ZFP36* as potential target genes in the development of OA-associated synovitis.** A** Protein–protein interaction network of the hub genes. Red represents upregulation and blue represents downregulation. **B** Venn diagram for screening *JUN* and *ZFP36*. **C** Distribution of *JUN* and *ZFP36* expression in the internal datasets. **D** Diagnostic ROC curves of *JUN* and *ZFP36* for OA-associated synovitis. **E** GSEA analysis of *JUN* and *ZFP36*. DEGs, differentially expressed genes; ROC, receiver operating characteristic; GSEA, gene set enrichment analysis; ^**^P < 0.01, ^***^P < 0.001 versus the control group

The PPI network constructed using the set of 183 DEGs is shown in Fig. 3A. A total of 50 hub genes were identified and ranked from the PPI network with the maximal clique centrality (MCC) algorithm of the Cytohubba plugin in Cytoscape. The size of each node was proportional to its degree of betweenness centrality. The color of the node was related to its logFC value. The MCC rank score of hub genes is shown in Additional file 2: MCC rank score. Interestingly, *JUN* and *ZFP36* were screened in the intersection of DEGs, hub genes, and ferroptosis-related genes (Fig. 3B). The mRNA expression levels of *JUN* and *ZFP36* were significantly reduced in OA-associated synovium (^***^P < 0.001, ^**^P < 0.01, respectively) (Fig. 3C). The ROC curve analysis suggested that *JUN* and *ZFP36* expression has a predictive value in the development of synovitis during the OA process, with the maximum AUC value reaching 0.78 and 0.74, respectively (Fig. 3D). The GSEA was performed to determine the differences in biological characteristics between patients with high expression and low expression of *JUN* or *ZFP36* (Fig. 3E). The low-expression *JUN* group was mainly enriched in biological processes including nucleotide excision repair, oxidative phosphorylation, mismatch repair, and pyrimidine metabolism. In contrast, the low-expression *ZFP36* group was mainly enriched in biological processes such as the B cell receptor signaling pathway, apoptosis, T cell receptor signaling pathway, and oxidative phosphorylation. The GSEA results are shown in Additional file 3: GSEA results of *JUN* and *ZFP36*.

### *JUN* and *ZFP36* expression correlated with mast cell activation and macrophage transformation during OA-associated synovitis

**Fig. 4:**
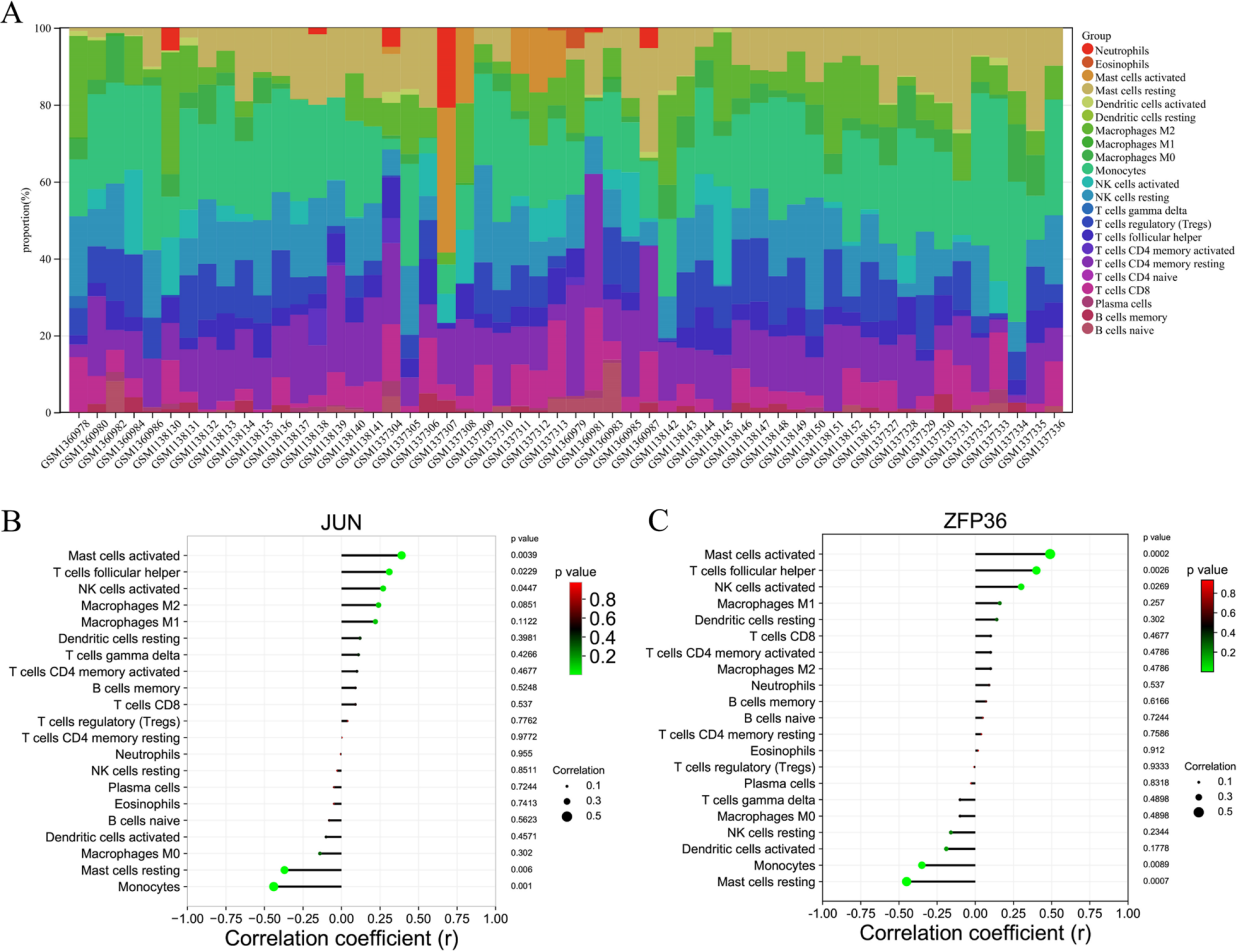
Immune cell infiltration and correlation with *JUN* and *ZFP36*.** A** Landscape of the proportion of 22 immune cells based on the internal datasets. **B** Correlation of immune cell infiltration with *JUN*. **C** Correlation of immune cell infiltration with *ZFP36*

The abundance distribution of 22 immune cell types in each sample is shown through stacked histograms, as illustrated in Fig. 4A. The distinct colors used in the histograms correspond to the various immune cell types. The height of each color indicates the percentage of cells present in the sample, with a cumulative total of 100%. The immune cell population in the synovium was heterogeneous, with predominant macrophages, monocytes, mast cells, dendritic cells, and T cells (Fig. 4A). The results of immune cell infiltration analysis are shown in Additional file 4: Immune infiltration analysis. Additionally, our findings indicate that the decreased mRNA expression levels of *JUN* and *ZFP36* were positively correlated with the activation of mast cells, follicular helper T cells, activated natural killer cells, and M2 macrophages, but were negatively correlated with monocytes, resting mast cells, M0 macrophages, and activated dendritic cells (Fig. 4B and C).

### External dataset validation for *JUN* and *ZFP36* supported the results of the internal dataset

**Fig. 5:**
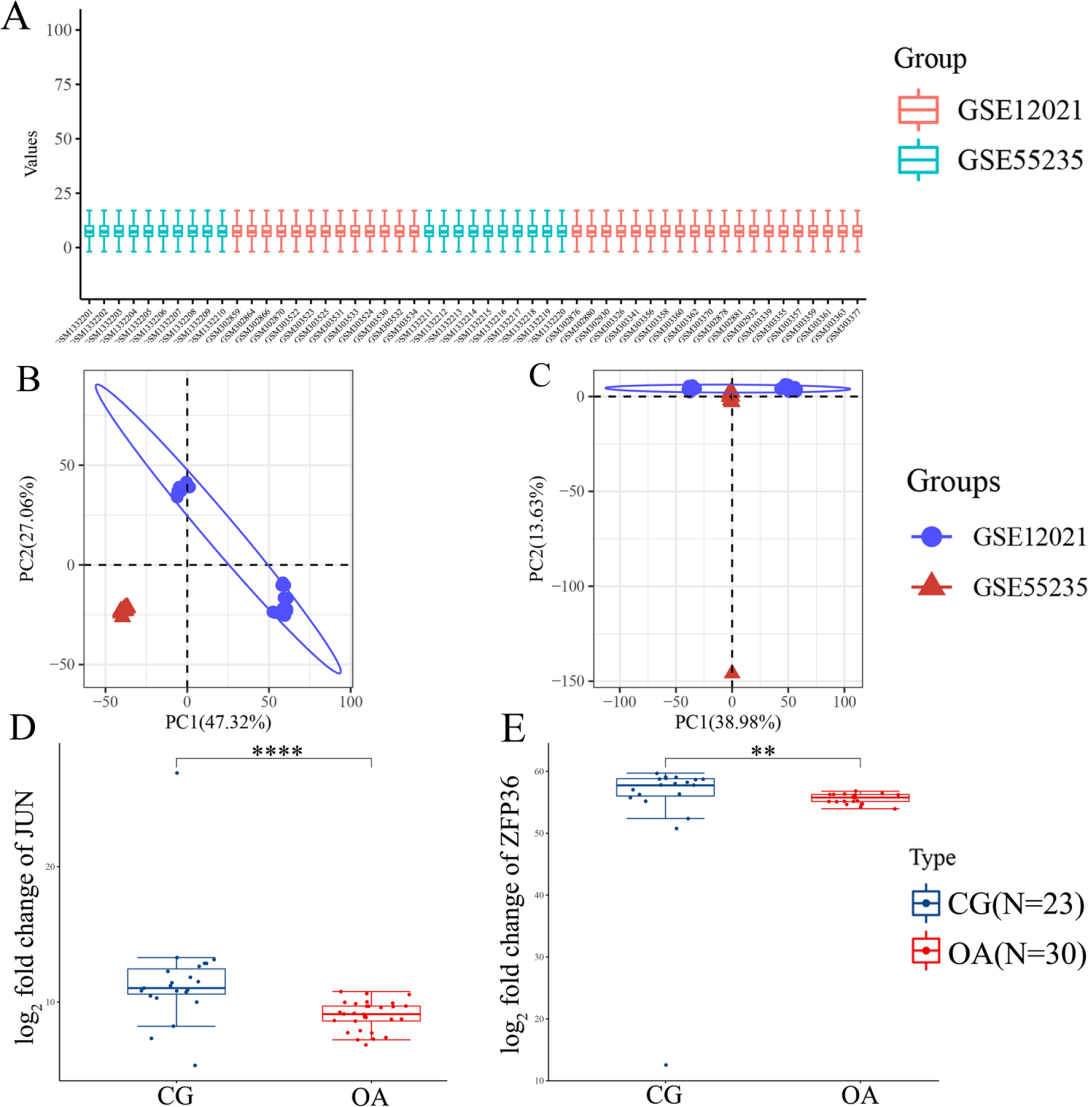
Distribution of *JUN* and *ZFP36* expression in the external datasets for validation. **A** Normalization of the GSE12021 and GSE55235 datasets. **B** PCA analysis before batch effect removal. **C** PCA analysis after batch effect removal. **D** Distribution of *JUN* expression in the external dataset. **E** Distribution of *ZFP36* expression in the external dataset. PCA, principal component analysis. ^**^P < 0.01, ^****^P < 0.0001 versus the control group

The external datasets, namely GSE12021 and GSE55235, were used to validate the expression of *JUN* and *ZFP36* mRNAs. Homogeneity was achieved in the external datasets (Fig. 5A–C). The results of both the external and internal datasets were consistent, indicating a reduction in the expression of *JUN* and *ZFP36* proteins in the OA-associated synovium as compared to that in the healthy synovium (Fig. 5D and E, ^**^P < 0.01, ^****^P < 0.0001).

### Single-cell RNA-seq profiling revealed *JUN* and *ZFP36* expression in different cell clusters

**Fig. 6:**
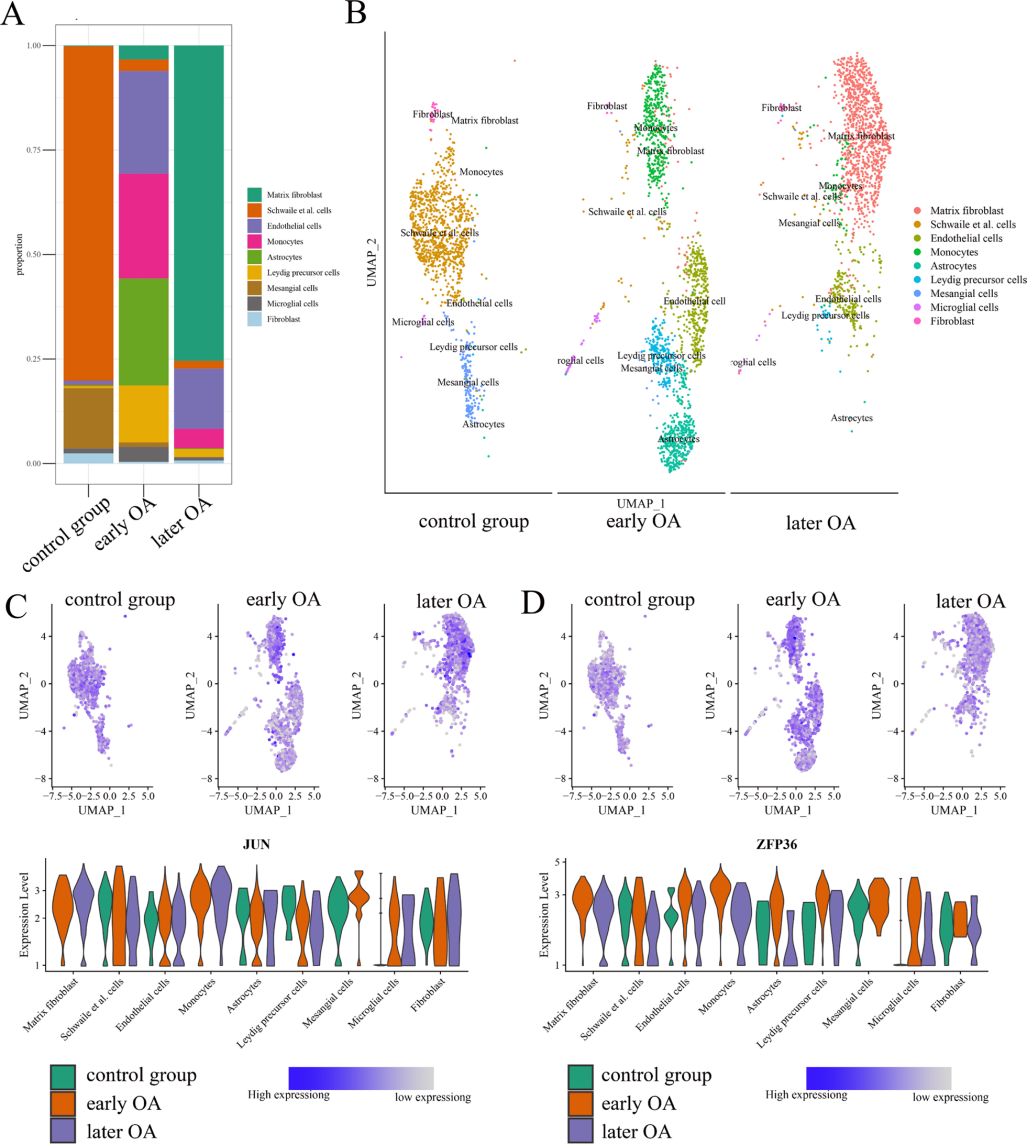
Analysis of *JUN* and *ZFP36* expression in different cell clusters through single-cell RNA-seq profiling. **A** The evolution of the proportion of each cell cluster within the synovium. **B** Landscape of each cell cluster with OA progression. **C** *JUN* expression in each cell cluster.** D** *ZFP36* expression in each cell cluster

GSM5362559, GSM5362560, and GSM5362561 were used as representative of advanced OA, early OA, and a control group, respectively. These were obtained from the GSE176308 dataset and subjected to single-cell RNA-seq profiling. Prior to the identification of cell clusters, a requisite quality control process was performed for the cells, which included the assessment of featured RNA quantity, RNA count quantity, mitochondrial RNA percentage, elimination of batch effect, and screening of highly variable feature genes (Additional file 5: Quality control and pretreatment of GSE176308). The synovium is composed of diverse cell clusters, including fibroblasts, Schwalie cells, endothelial cells, monocytes, astrocytes, Leydig precursor cells, mesangial cells, microglial cells, and matrix fibroblasts. The proportions of cell clusters exhibited a significant variation with the progression of OA-associated synovitis. For example, the proportion of matrix fibroblasts showed a significant increase, whereas the proportion of monocytes and endothelial cells showed an initial increase followed by a subsequent decline (Fig. 6A and B). The violin diagrams in Fig. 6C and D show the expression of *JUN* and *ZFP36* across various cell clusters and OA stages. Unexpectedly, variations in *JUN* and *ZFP36* expression across specific cell clusters (matrix fibroblasts, endothelial cells, and fibroblasts) were inconsistent with the results of the microarray analysis. This difference can be primarily attributed to changes in the proportion of cell clusters rather than individual cells.

### Synovitis development accelerated OA progression

**Fig. 7:**
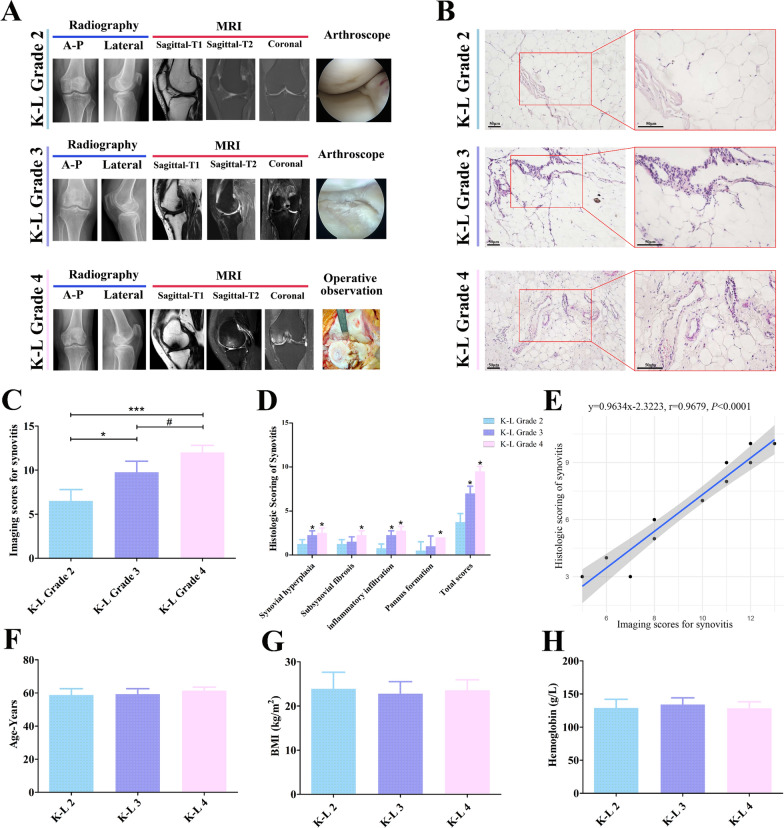
Imaging scores of synovitis are consistent with its pathological features from clinical synovial samples. **A** Preoperative X-ray and MRI examinations and intraoperative imaging of each group. **B** HE staining of synovial samples. **C** Imaging scores of synovitis. **D** Histologic scores of synovitis. **E** Univariate linear regression analysis of radiographic and histologic scores of synovitis. **F** Age distribution of patients. **G** BMI of patients. **H** Hemoglobin level of patients. HE, hematoxylin and eosin; BMI, body mass index; ^*^P < 0.05, ^***^P < 0.001 versus K–L Grade 2; ^#^*P* < 0.05 versus K–L Grade 3

Four patients per group were recruited for this study. The preoperative X-ray and MRI as well as the intraoperative images are shown in Fig. 7A. The synovium samples collected during surgery were subjected to HE staining (Fig. 7B). With the progression of synovitis, the synovium exhibited pannus formation, inflammatory infiltration, fibrosis, and hyperplasia. Subsequently, the severity of synovitis was radiologically and histologically quantified in accordance with previous methods [23, 24]. Both radiographic and histological scores significantly increased with the aggravation of K–L grade (Fig. 7C and D, ^*^P < 0.05, ^***^P < 0.001 versus K–L Grade 2; ^#^*P* < 0.05 versus K–L Grade 3). The univariate linear regression analysis demonstrated that these two scoring systems were significantly correlated in OA progression (Fig. 7E, P < 0.0001). The results of age, body mass index (BMI), and preoperative hemoglobin suggested that the baseline characteristics of each group were comparable (Figs. 7F–H).

### Iron overload and its associated adverse events could be risk factors for the deterioration of synovitis

**Fig. 8:**
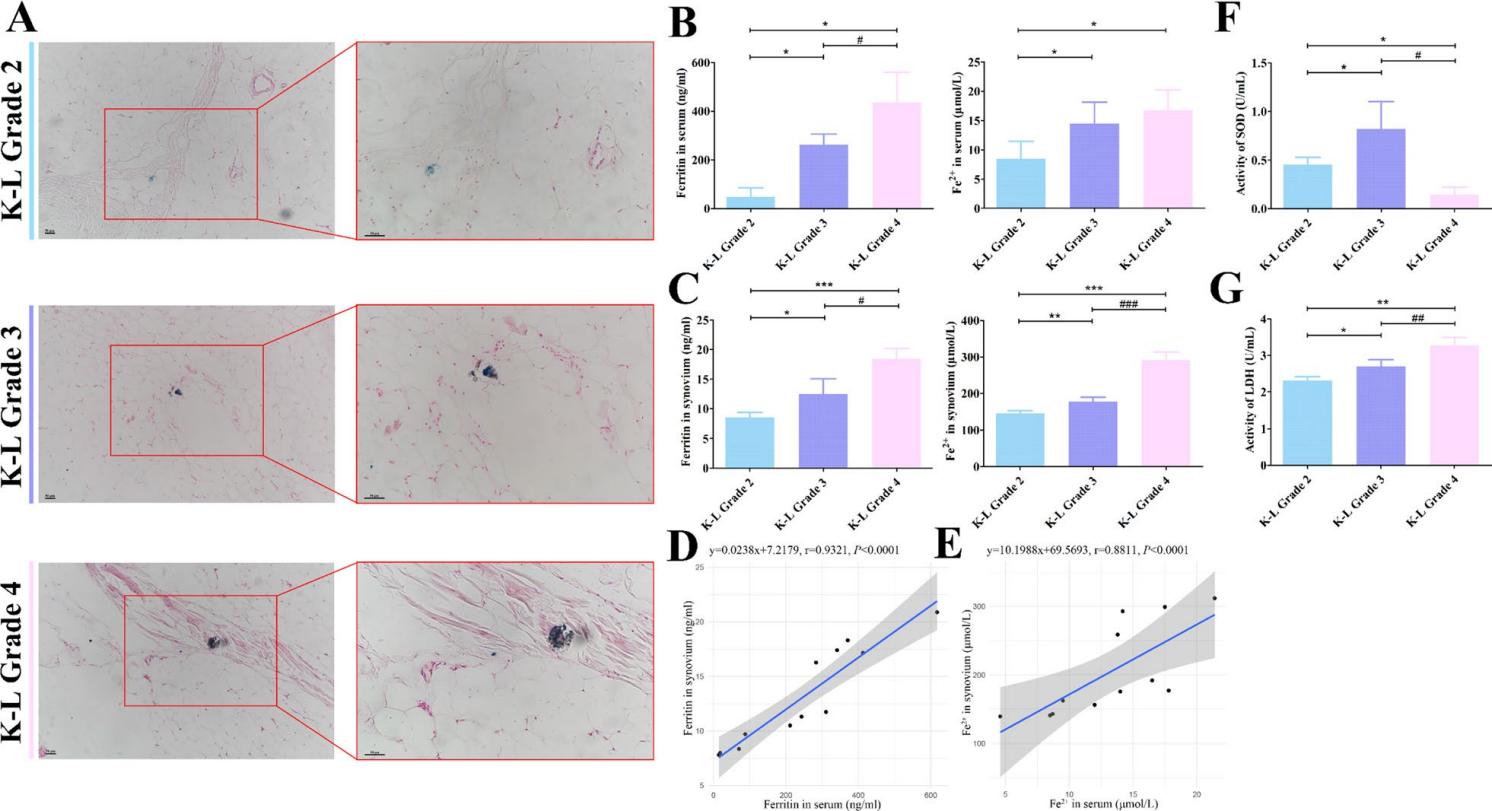
Synovium facilitates the conversion of iron from a systemic distribution to a localized distribution. **A** Prussian Blue staining of the synovium. **B** Serum levels of ferritin and Fe^2+^. **C** Ferritin and Fe^2+^ levels in the synovium. **D** Univariate linear regression analysis between ferritin levels in serum and synovium. **E** Univariate linear regression analysis between Fe^2+^ levels in serum and synovium. **F** Activity of SOD. **G** Activity of LDH. SOD, superoxide dismutase; LDH, lactate dehydrogenase; ^*^P < 0.05, ^**^P < 0.01, ^***^P < 0.001 versus K–L Grade 2; ^#^*P* < 0.05, ^##^*P* < 0.01, ^###^*P* < 0.001 versus K–L Grade 3

Prussian Blue staining revealed the presence of iron accumulation at the site of synovial pannus formation, which corresponded with OA progression (Fig. 8A). A notable upward trend in the serum levels of ferritin and Fe^2+^ was observed with the progression of K–L grade (Fig. 8B, ^*^P < 0.05 versus K–L Grade 2; ^#^*P* < 0.05 versus K–L Grade 3). We also observed an increasing trend of ferritin and Fe^2+^ levels in the synovium with the progression of K–L grade (Fig. 8C, ^*^P < 0.05, ^**^P < 0.01, ^***^P < 0.001 versus K–L Grade 2; ^#^*P* < 0.05, ^###^*P* < 0.001 versus K–L Grade 3). A strong correlation was observed between systemic and localized iron overload as reflected by the levels of ferritin and Fe^2+^ (Fig. 8D and E). These findings indicate a potential association between iron overload and the progression of synovitis. To confirm our findings, we detected the activity of superoxide dismutase (SOD) and lactate dehydrogenase (LDH) in the synovium to determine the effects of iron overload on synovitis. Remarkably, SOD activity exhibited an initial increase followed by a subsequent decrease (Fig. 8F, ^*^P < 0.05 versus K–L Grade 2; ^#^*P* < 0.05 versus K–L Grade 3). The augmented activity of LDH in K–L Grade 4 as compared to that in K–L Grade 2 and 3 was discernible (Fig. 8G, ^*^P < 0.05, ^**^P < 0.01 versus K–L Grade 2; ^##^*P* < 0.01 versus K–L Grade 3).

### Synovitis development may be associated with the differential expression of *JUN* and *ZFP36* induced by iron overload

**Fig. 9:**
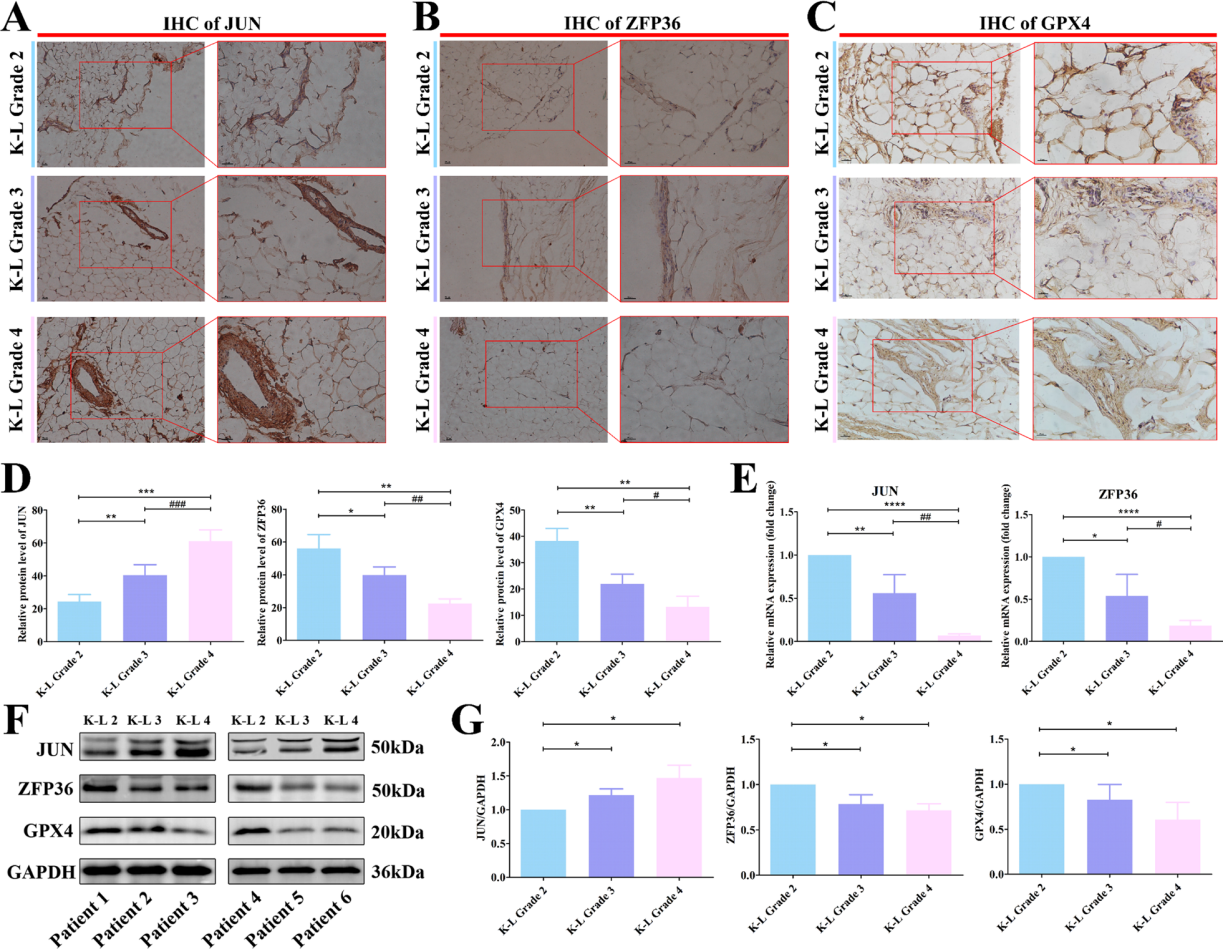
Validation of *JUN* and *ZFP36* expression at the protein level from clinical synovial samples. **A** IHC of *JUN*. **B** IHC of *ZFP36*. **C** IHC of *GPX4.* **D** Relative protein levels of *JUN*, *ZFP36* and *GPX4* from IHC. **E** Relative mRNA expression levels of *JUN* and *ZFP36*. **F** Western blotting assay of *JUN*, *ZFP36*, *GPX4*, and *GAPDH.* **G** Statistical analysis of the results of western blotting assay. IHC, immunohistochemistry. ^*^P < 0.05, ^**^P < 0.01, ^***^P < 0.001, ^****^P < 0.0001 versus K–L Grade 2; ^#^*P* < 0.05, ^##^*P* < 0.01, ^###^*P* < 0.001 versus K–L Grade 3

Immunohistochemistry (IHC) findings suggested an upward trend of *JUN* expression, downward trends of *ZFP36* and *GPX4* (Fig. 9A–D, ^*^P < 0.05, ^**^P < 0.01, ^***^P < 0.001 versus K–L Grade 2; ^#^*P* < 0.05, ^###^*P* < 0.001 versus K–L Grade 3). This finding is noteworthy and potentially surprising because of the lack of consistency between the transcript and protein levels of *JUN*. It was further corroborated by the results of qRT-PCR and western blotting assay. The qRT-PCR findings indicated a significant reduction in the transcript levels of *JUN* and *ZFP36* (Fig. 9D, ^*^P < 0.05, ^**^P < 0.01, ^****^P < 0.0001 versus K–L Grade 2; ^#^*P* < 0.05, ^##^*P* < 0.01 versus K–L Grade 3). However, the protein expression of *JUN* exhibited a significant increase, whereas that of *ZFP36* continued to exhibit a decline (Fig. 9E and F, ^*^P < 0.05 versus K–L Grade 2). The protein level of *GPX4* also significantly decreased with the aggravation of OA.

## Discussion

OA is a slowly progressing degenerative disease primarily responsible for nontraumatic disability in the elderly population [25]. The hypothesis of OA pathogenesis has been expanded to include an initial injury or changes in joint appendages that trigger an imbalance of the cartilage microenvironment, ultimately resulting in cartilage degeneration [26, 27]. Synovial inflammation is believed to contribute to radiographic distortion and pain deterioration in OA [28]. Here, we reveal that the synovium is a sensitive tissue for mapping the negative effects of systemic iron overload in OA progression. The genes *JUN* and *ZFP36* were screened from the DEGs of synovitis and could be considered potential targets for relieving iron overload. However, some discrepancies were observed in the transcription and expression of the genes *JUN* and *ZFP36* from our validation results. Moreover, the expression levels of *JUN* and *ZFP36* exhibited heterogeneity across the cell clusters of the synovium and strongly correlated with immune cell infiltration.

The degree of synovitis on MRI is associated with both gross appearance of the synovium and histological evidence, and this may have clinical significance related to the pain and radiographic deterioration of OA [29]. The thickening of the synovium as observed in MRI is attributed to microscopic characteristics such as fibrin deposition, fibrosis, and pannus formation. This phenomenon may be caused by the overexpression of type III collagen, which alters the balance between tissue formation and degradation, and it is associated with the sensitization of pain in individuals with knee OA [30]. Synovitis also represents an adaptive response to systemic or localized iron overload [31]. Our results revealed that iron overload (elevated levels of ferritin and Fe^2+^) existed in the synovium derived from the suprapatellar region with pain. Consequently, the mitigation of iron overload could serve as a viable strategy to relieve joint pain resulting from synovitis. Nonetheless, the present concept remains deficient regarding forecasting and substantiating potential targets and pathways, thereby causing apprehensions related to potential unfavorable and unresolved outcomes.

In the present study, we screened and identified two possible target genes (*JUN* and *ZFP36*). The gene *JUN* encodes the *c-Jun* protein, which is an oncogenic transcription factor and can regulate glucose metabolism [32, 33]. *O*-linked β-N-acetylglucosamine glycosylated (*O*-GlcNAcylated) c-*Jun* increases the synthesis of glutathione to inhibit ferroptosis as a resistance mechanism against iron overload [34]. *ZFP36* is a member of the zinc finger protein family and is characterized by its two tandem repeat CCCH zinc finger domains [35]. Thus, *ZFP36* destabilizes target mRNAs by binding to 3ʹ-UTR AREs and recruiting dealkylation and degradation factors to perform post-transcriptional regulation, which is crucial in lipid peroxidation reactions during ferroptosis [36, 37]. The lower transcript levels of *JUN* and *ZFP36* were predicted and validated in OA-associated synovitis, and this approach has high diagnostic efficacy for OA-associated synovitis. Moreover, this study revealed that changes in *JUN* and *ZFP36* expression were correlated with those in *GPX4* expression, a marker of ferroptosis. Considering the role of *JUN* and *ZFP36* as transcription factors, it is reasonable to speculate that they may regulate *GPX4* expression to influence the response of synovial tissue to iron overload.

Although the *JUN* gene has been recently screened in other diseases, these studies are yet to be done and provide a deeper understanding of the mechanism [38]. In the present study, we observed an inconsistency between the expression and transcription of *JUN* in synovitis. The increased expression of the *JUN* protein can trigger ferroptosis through the *JNK-JUN-NCOA4* mediated ferritinophagy pathway [39]. Conversely, the *JUN* protein can elevate glutathione synthesis and impede ferroptosis through *O*-GluNAcylation at the Ser73 site [40]. Moreover, the low transcription level of the *JUN* gene can be considered a negative feedback regulation triggered by iron overload in synovitis. Therefore, the use of posttranscript modification or *O*-GluNAcylation presents a promising approach to manage OA in the future, as it may effectively mitigate synovial iron overload.

The synovium is also a multicellular component tissue containing fibroblasts, immune cells, vascular endothelial cells, and nerve-related cells as revealed by single-cell RNA-seq profiling and immune cell infiltration analysis. Fibroblasts are the main cellular component of the synovium and function as the primary factor of synovitis pathology [41]. With the progression of OA, fibroblasts are gradually replaced by matrix fibroblasts. Although the increased number of matrix fibroblasts compensates for the absence of other cells, it may not allow the synovium to perform its original function. Changes in synovial blood composition and the heterogeneity of resident monocytes are also involved in the maintenance of synovitis [42, 43]. Endothelial cell-derived vascular endothelial growth factor has significant involvement in the pathogenesis of OA. Our results suggest that the deficiency in the transcript levels of *JUN* and *ZFP36* may induce an imbalance in mast cell activation and macrophage polarization. The presence of nerve tissue also makes the synovium sensitive to mechanical stimulation and can precisely localize pain [44]. Collectively, these findings suggest a noteworthy diversity in cellular subpopulations and intercellular communication networks within the synovitis tissue, thus necessitating additional studies for characterization and comprehension.

Several previous studies and bioinformatics analyses of OA synovitis have been reported since 2023. These studies were dedicated to exploring key target genes in the development of OA synovitis from the perspective of cuproptosis, ferroptosis, potential drugs, and aging [45–48]. Based on this, we performed a series of studies on baseline, imaging, histology, and indicators of iron overload to illustrate the iron sensitivity and pathological mapping ability of the synovium. Finally, we verified changes in *JUN* and *ZFP36* expression at the gene and protein levels. Furthermore, GSE55235 (10CG vs. 10OA) and GSE55457 (10CG vs. 10OA) were included in all of the above-mentioned articles [45–48]. We added an additional three microarray datasets (GSE46750:12CG vs.12OA, GSE56409:5CG vs. 5OA, and GSE12021:13CG vs. 20OA) to enhance the generalizability of our results. Therefore, our study has 37.4% similarity and 62.6% uniqueness compared to the studies discussed above.

Although the present study has revealed important findings regarding the relationship between iron overload and OA-associated synovitis, it still has some limitations. First, more real-world sequencing data are required to improve the predictive power of the obtained results. Second, future studies should collect and group samples from KL0 to KL4 to reflect the dynamic progression of OA. Third, the underlying regulatory and downstream mechanisms of *JUN* and *ZFP36* in synovitis require further studies.

In conclusion**,** the present study elucidated the sensitivity of the synovium in mapping the adverse effects of systemic iron overload on OA progression. Two genes, i.e., *JUN* and *ZFP36*, were identified by screening the DEGs associated with synovitis. *JUN* and *ZFP36* expression levels showed variations among synovial cell clusters and were highly associated with the infiltration of immune cells. We conclude that *JUN* and *ZFP36* could be the potential target genes to attenuate iron overload in patients with OA.

### Supplementary Information

**Additional file 1.** Enrichment analysis of KEGG and GO.

**Additional file 2.** MCC rank score.

**Additional file 3.** GSEA results of *JUN* and *ZFP36*.

**Additional file 4.** Immune infiltration analysis.

**Additional file 5.** Quality control and pretreatment of GSE176308.

## Data Availability

The datasets generated and/or analyzed during the current study are available in the Gene Expression Omnibus database (https://www.ncbi.nlm.nih.gov/geo/) (Accession Numbers: GSE46750, GSE55457, GSE56409, GSE55235, GSE12021, and GSE176308).
